# Predicting Axillary Metastasis of Breast Cancer Patients with MRI Relaxometry

**DOI:** 10.3390/diagnostics15020188

**Published:** 2025-01-15

**Authors:** Roxana Pintican, Radu Fechete, Delia Ioana Radutiu, Manuela Lenghel, Ioana Bene, Carolina Solomon, Cristiana Ciortea, Anca Ciurea

**Affiliations:** 1 Department of Radiology, “Iuliu Hatieganu” University of Medicine and Pharmacy, 400347 Cluj-Napoca, Romania; deliaioanaradutiu@gmail.com (D.I.R.); manu_2416@elearn.umfcluj.ro (M.L.); ioanabene@elearn.umfcluj.ro (I.B.); carolina.solomon@elearn.umfcluj.ro (C.S.); cristianaciortea@yahoo.com (C.C.); ancaciurea@elearn.umfcluj.ro (A.C.); 2 Department of Radiology, Prof. Dr. Ion Chiricuta Oncology Institute, 400015 Cluj-Napoca, Romania; 3 Institute for Interdisciplinary Research in Bio-Nano-Science, Babes-Bolyai University, INSPIRE Platform, 400347 Cluj-Napoca, Romania; 4 Faculty of Material and Environmental Engineering, Physics and Chemistry Department, Technical University of Cluj-Napoca, 400114 Cluj-Napoca, Romania; 5 Department of Radiology, County Emergency Hospital, 400347 Cluj-Napoca, Romania

**Keywords:** axillary metastasis, breast cancer, N stage, relaxometry, axillary MRI

## Abstract

**Background:** Breast cancer is a leading cause of cancer-related mortality among women worldwide. Accurate staging, including the detection of axillary metastases, is vital for treatment planning. This study evaluates the efficacy of MRI relaxometry as a diagnostic tool for axillary lymph node metastases in breast cancer patients. **Methods:** A prospective study was conducted on 67 consecutive breast cancer patients. Relaxometry parameters, including T2Max, T2Min, and 1HAv, were assessed using 1.5 Tesla MRI. All axillary metastases were histologically confirmed using core-needle biopsy or surgical specimens. Statistical analyses included ROC curves, chi-square tests, and multivariate analysis to determine correlations between imaging findings and pathological results. **Results:** Significant associations were found between T2Min-ipsilateral (*p* = 0.018), 1HAv-ipsilateral (*p* = 0.003), and axillary metastases. ROC analysis demonstrated that T2Min-ipsilateral and 1HAv-ipsilateral have modest to acceptable discriminatory abilities (AUC = 0.681 and AUC = 0.740, respectively). Combined clinical and imaging models enhanced diagnostic accuracy (AUC = 0.749). **Conclusions:** MRI relaxometry improves the detection of axillary metastases in breast cancer, particularly when integrated with clinical and pathological evaluations.

## 1. Introduction

Axillary lymph node status is a critical factor in the staging, treatment planning, and prognosis of breast cancer [1,2]. The presence or absence of metastases in these nodes informs therapeutic decisions, including the need for systemic therapy, and provides key prognostic information regarding disease progression and overall survival. Axillary metastases are associated with a higher risk of distant spread and recurrence with a 5-year overall survival rate of 85.8% compared to 98.8% in axillary lymph node-negative patients [3]. All current guidelines, including those from the NCCN, ESMO, and ASCO, recommend the evaluation of axillary lymph nodes prior to any treatment, in order to guide further treatment [4,5,6]. Furthermore, nodal status serves as a prediction marker, alone or in combination with other factors, such as the tumor size or distant metastasis [7,8].

The pathological evaluation of axillary lymph nodes remains the gold standard for diagnosis, but despite the clinical significance, the challenge remains in reliably differentiating metastatic from benign nodes using non-invasive techniques.

Conventional imaging methods such as ultrasound (US) and standard magnetic resonance imaging (MRI) have been extensively employed to assess axillary lymph nodes. US is widely available, cost-effective, and often used as the first-line modality for axillary evaluation. However, its accuracy is operator-dependent, and its sensitivity can be suboptimal in detecting micro-metastases. The overall reported sensitivity of US in depicting the axillary metastases is between 20 and 94%, with pooled estimates for all studies of 50%. US may fail to detect small or deep-seated metastatic deposits, while biopsy, although definitive, is invasive and associated with potential complications, including sampling errors and false negatives. Compared to US fine needle aspiration, core needle biopsy shows higher accuracy in the detection of metastasis, but still has a false negative rate up to 25% estimated for all studies, especially in patients with invasive lobular carcinoma or after neoadjuvant treatment [9,10,11].

These shortcomings highlight the need for advanced, non-invasive methods capable of providing reliable and accurate assessment of axillary lymph node involvement.

MRI, on the other hand, offers superior soft tissue contrast and detailed anatomical imaging, making it more effective for visualizing the axilla. Yet, even with advanced MRI techniques, the diagnostic accuracy for axillary metastases remains limited by overlap in imaging characteristics between metastatic and reactive nodes. Some studies report similar sensitivity with US, while others suggest lower diagnostic accuracy in axillary metastasis [10,12,13]. Emerging methods, such as positron emission tomography (PET) and radiomics, show promise but are not routinely accessible due to high costs and complexity.

MRI relaxometry represents a novel, quantitative imaging approach that assesses tissue properties by measuring T2 relaxation times and proton signal intensities and does not require contrast media. By quantifying relaxation parameters, studies identified patients with malignant breast tumors and patients with complete response after neoadjuvant treatment [14,15]. This technique provides unique insights into the microstructural environment of tissues and may enable the differentiation of malignant and benign lymph nodes based on their biophysical properties. MR relaxometry offers a non-invasive, objective approach to axillary staging, potentially enhancing diagnostic precision without the risks and discomfort associated with traditional methods. This advancement could improve clinical decision-making, reduce the need for invasive procedures, and ultimately lead to better patient outcome

The primary objective of this study is to assess the effectiveness of MRI relaxometry in detecting axillary metastases among breast cancer patients. Additionally, it seeks to evaluate the diagnostic accuracy of MRI relaxometry by comparing its findings directly with histopathological results, the current gold standard.

## 2. Materials and Methods

The study was conducted as an observational, retrospective, cross-sectional analysis between November 2023 and June 2024 at the Clinical Emergency Hospital, Cluj-Napoca, on 125 patients. The MR relaxometry sequences were previously acquired for a study focused on the breast tumor characterization, for which all the participant signed a written consent. For the current IRB-approved study, the committee waived the need for a second written consent.

The inclusion criteria required patients with proven breast cancer that have undergone MRI relaxometry with sequences suitable for T2 mapping prior to any treatment and histopathological confirmation of axillary node status. Both core-needle biopsies and surgical specimens of the axilla and lymph nodes were included in the analysis. Patients were excluded if they had benign breast lesions, incomplete data or axillary images, or if imaging was performed during or after curative treatment. Patients with benign breast tumors were excluded from the study because they could not have axillary metastases. Patients undergoing therapy or who completed therapy were excluded because the characteristics of the lymph nodes would have been altered and would no longer reflect the basic relaxometry properties. Data collected included patient age, tumor type, Nottingham grade, tumor receptor status (ER, PR, HER2), and quantitative MRI parameters (T2Max, T2Min, T2Av, 1HMax, 1HMin, and 1HAv) measured ipsilaterally and contralaterally.

MRI was performed using a 1.5 Tesla machine with relaxometry sequences at specific echo times (TE1 = 50 ms; TE2 = 100 ms; TE3 = 150 ms; TE4 = 200 ms; TE5 = 250 ms). A specialized radiologist with four years of experience analyzed the images, delineating regions of interest (ROIs) for quantitative assessment. Statistical analyses were conducted using MedCalc software (version 19.2.6). Continuous variables were compared using *t*-tests, while categorical variables were analyzed with chi-square or Fisher’s exact tests. The *t*-test was used to compare continuous variables between two groups (with and without axillary metastasis). For categorical variables, the chi-square test was used to assess the association between categorical variables, when the expected frequencies were sufficiently large (>5). Fisher’s exact test was applied as an alternative, particularly for small sample sizes (<5). Receiver operating characteristic (ROC) curves were used to evaluate the diagnostic performance of significant imaging parameters in identifying axillary metastasis. Multivariate regression analysis was conducted to identify independent predictors of axillary metastasis (such as tumor size, ki67% proliferation index, or hormonal receptors). Statistical significance was set at *p* < 0.05.

## 3. Results

### 3.1. Patient Demographics and Tumor Characteristics

The study analyzed data from 67 female patients aged between 31 and 87 years, with a mean age of 56.42 ± 11.58 years for those without axillary metastases and 57.14 ± 10.17 years for those with metastases. The difference in age between these two groups was not statistically significant (*p* = 0.481). All patients were female, reflecting the high prevalence of breast cancer in this demographic.

Most of the tumors were invasive ductal carcinomas (77.3%) and when tested between groups, a tendency towards statistical significance was observed (*p* = 0.074), indicating a higher frequency of invasive ductal carcinomas in patients with axillary metastases (90.7% vs. 68.7%) and a higher frequency of invasive lobular carcinomas in patients without metastases (13.4% vs. 2.3%).

The Nottingham grading system revealed a statistically significant correlation with axillary metastases. Patients with grade 3 tumors exhibited a higher likelihood of metastasis, as compared to those with grade 1 or grade 2 tumors (*p* = 0.042). Analysis of receptor status showed that ER positivity was significantly associated with axillary metastases (*p* = 0.019), while no significant correlation was observed with PR status (*p* = 0.165) or HER2 status (*p* = 0.076).

The Ki-67 proliferation index values did not show a statistically significant association with axillary metastases. Patients were then grouped based on a cut-off value of 20%, and the analysis revealed no substantial differences between metastatic and non-metastatic groups (*p* = 0.121) (Table 1).

### 3.2. Relaxometry Imaging Findings

MRI relaxometry parameters were obtained for ipsilateral and contralateral lymph nodes of all patients. Ipsilateral measurements of T2Min showed a significantly higher mean value in metastatic nodes (73.94 ± 53.32 ms) compared to benign lymph nodes (41.99 ± 53.30 ms), with a *p*-value of 0.018. Similarly, 1HAv-ipsilateral values were significantly lower in metastatic nodes (148.28 ± 50.68 arbitrary units) compared to benign nodes (186.79 ± 44.46 arbitrary units, *p* = 0.003).

**Figure 1:**
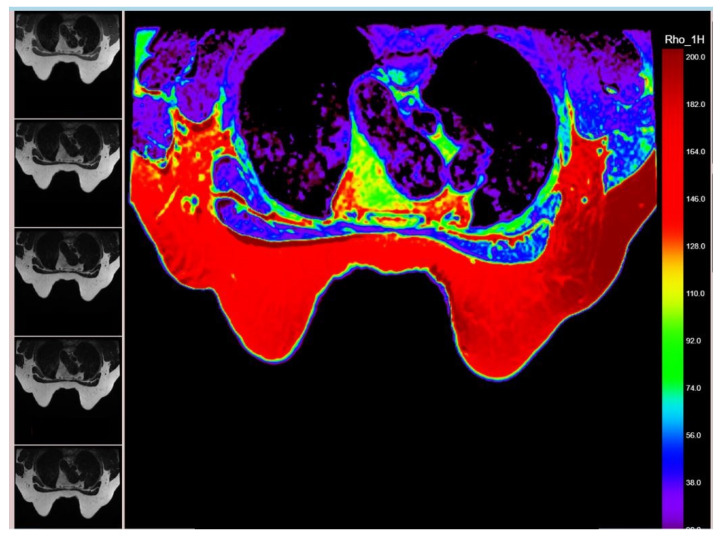
MR proton density map derived based on the 5th T2WI relaxometry sequences on the left side; the map is highlighting a micro-metastasis on an axillary lymph node on the left side.

Contralateral measurements generally showed weaker correlations. However, 1HAv-contralateral values were still statistically significant, with lower signal intensities observed in metastatic cases (*p* = 0.007). Parameters such as T2Av and T2Max did not demonstrate significant differences between metastatic and benign nodes, suggesting that T2Min is the most reliable imaging marker in this study (Table 2 and Figure 1).

### 3.3. Correlation Between Tumor Type and Axillary Status

A chi-square test evaluating the relationship between tumor type (invasive ductal carcinoma, invasive lobular carcinoma, and papillary carcinoma) and axillary metastases revealed no statistically significant correlation (*p* = 0.294). The majority of cases involved invasive ductal carcinoma (92.5%), which was present across both metastatic and non-metastatic groups.

### 3.4. Multivariate Analysis of Imaging and Clinical Parameters

A multivariate analysis was conducted to determine the independent predictors of axillary metastases. Variables included in the analysis were T2Min-ipsilateral, 1HAv-ipsilateral, 1HAv-contralateral, Nottingham grade, and ER status. Among these, T2Min-ipsilateral emerged as the most significant predictor, with a *p*-value of 0.018. Nottingham grade also showed a strong association, with higher grades correlating with metastatic involvement.

**Figure 2:**
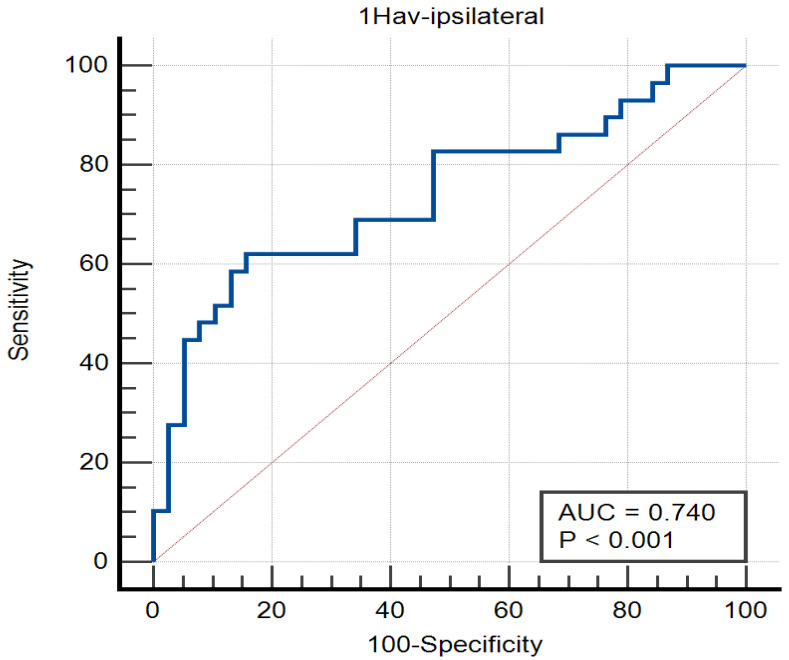
Graphical representation of the ROC curve for the variable “1Hav-contralateral” with *p* = 0.0002.

ROC curve analysis was performed to evaluate the diagnostic performance of relaxometry parameters. T2Min-ipsilateral demonstrated an area under the curve (AUC) of 0.681, indicating modest discriminatory ability (*p* = 0.010). In contrast, 1HAv-ipsilateral achieved a higher AUC of 0.740, reflecting acceptable diagnostic performance (*p* < 0.001). The contralateral parameter 1HAv-contralateral had an AUC of 0.677 (*p* = 0.008), showing moderate discriminatory capability (Figure 2).

### 3.5. Nomogram and Comparative Analysis

To further validate the predictive capability of MRI relaxometry, a nomogram was constructed using the significant variables from multivariate analysis. A combined diagnostic model incorporating T2Min-ipsilateral, Nottingham grade, and ER status achieved an improved AUC of 0.749, demonstrating the potential of an integrated approach. This model highlights the importance of combining imaging parameters with clinical and pathological data for optimal diagnostic accuracy.

**Figure 3:**
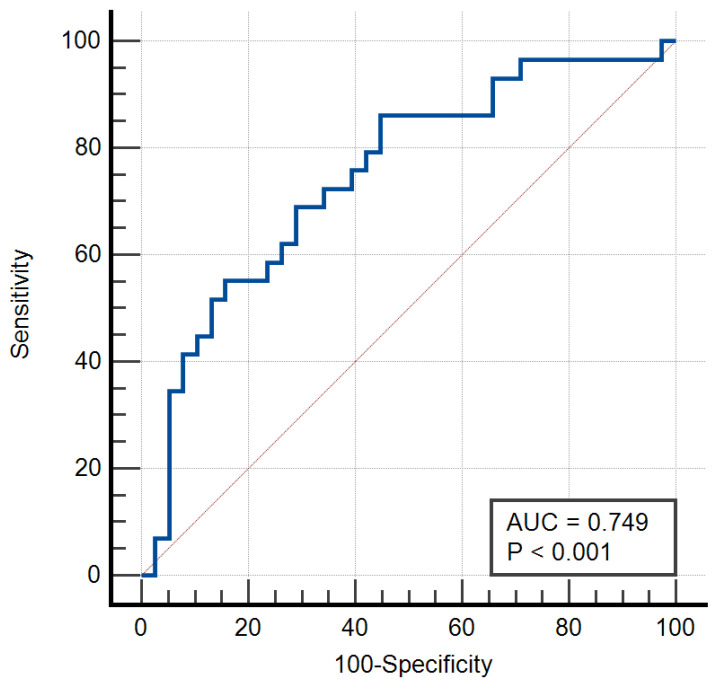
ROC curve representation of the nomogram including MR relaxometry and clinical parameters.

The nomogram achieved an AUC of 0.749, slightly outperforming individual imaging parameters such as T2Min-ipsilateral. Comparative analysis of ROC curves indicated that the combined approach is superior to using MRI relaxometry alone, underscoring the need for a multi-disciplinary diagnostic framework (Figure 3).

**Figure 4:**
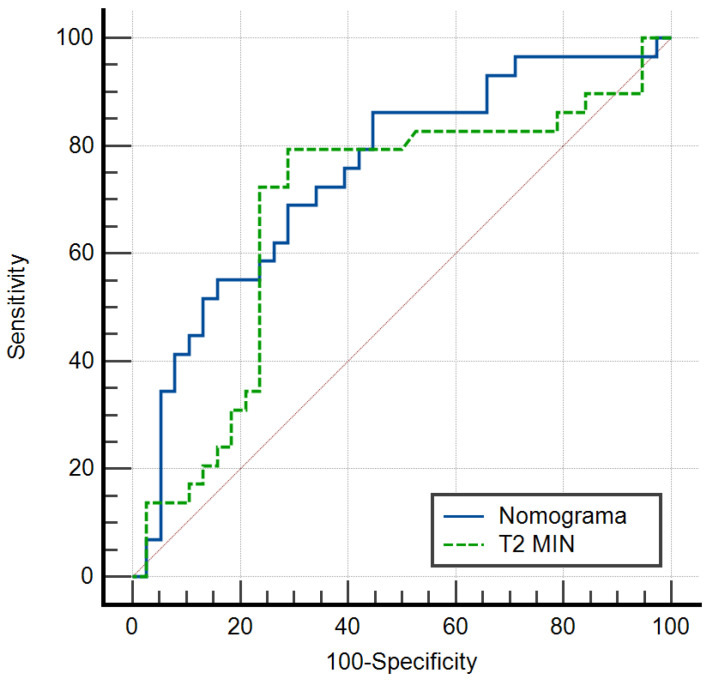
Comparison of the ROC curve of the nomogram and the variable “T2min-ipsilateral”.

Compared to the AUC = 0.681 of the parameter “T2MIN-ipsilateral”, a mixed relaxometry–clinical model is much more effective in detecting axillary metastases in patients diagnosed with breast cancer than using only MRI relaxometry (Figure 4).

## 4. Discussion

The aim of this study was to evaluate the efficacy of MRI relaxometry in distinguishing metastatic axillary lymph nodes from benign lymph nodes in breast cancer patients. Two quantitative MR relaxometry parameters (T2Min-ipsilateral and 1HAv-ipsilateral) achieved acceptable diagnostic performance, with AUCs between 0.68 and 0.74. The integration of imaging with clinical and pathological parameters improved diagnostic accuracy.

Previous studies have investigated various imaging modalities for evaluating axillary lymph nodes. US, widely used for axillary assessment, has demonstrated variable sensitivity and specificity, often limited by operator dependency and its inability to detect micrometastases reliably. Studies report high variability in terms of sensitivity, with values between 20 and 87%, with a pooled sensitivity for all studies up to 50% [16,17,18,19,20]. However, US remains the first line imaging modality used to assess the axilla, even if its accuracy is moderate [21].

MRI, as a non-invasive imaging technique, offers significant potential in improving diagnosis, particularly when evaluating axillary status in patients. Unlike traditional methods such as US and biopsy, which require invasive procedures or additional steps, MRI can provide detailed and accurate insights into lymph node involvement without the need for tissue sampling. This approach can streamline the diagnostic process, saving time and reducing patient discomfort. By potentially allowing for a more direct assessment of axillary status, MRI can facilitate quicker decision-making in treatment planning, improving the overall flow into patient management and enhancing clinical outcomes.

Conventional MRI techniques have shown superior soft-tissue contrast but struggle to differentiate between metastatic and reactive lymph nodes based on anatomical features alone. Studies have shown that the diagnostic accuracy of morphological features observed on MRI for detecting axillary metastases is limited. Suspicious MRI features associated with malignancy include cortical thickening, loss of the fatty hilum, a round shape, or a long-to-short axis ratio of less than 2. While studies have investigated diffusion-weighted MRI (DWI) and dynamic contrast-enhanced MRI (DCE-MRI) for axillary node assessment, their diagnostic accuracy remains suboptimal, with AUC values often falling in the moderate range. Among various quantitative and qualitative descriptors, perifocal edema—characterized by marked T2 prolongation in the fat surrounding a lymph node—has demonstrated the highest positive predictive value for malignancy [22,23,24,25,26].

Emerging technologies like PET imaging have introduced promising results but remain less accessible due to high costs and complexity. PET/CT is not yet sensitive enough for detecting primary breast cancer or evaluating axillary lymph nodes in early-stage breast cancer (stages I and II). Its accuracy is particularly low for identifying micrometastases, with sensitivity decreasing to 33% [26,27,28].

Recent advancements in radiomics and radio-genomics have shown promise in improving lymph node assessment in breast cancer. Radiomics analysis using T2-weighted fat suppression and diffusion-weighted imaging has demonstrated strong predictive performance for sentinel lymph node metastasis, achieving AUCs between 0.770 and 0.863, highlighting the potential of noninvasive imaging tools to enhance clinical decision-making. The study highlighted that incorporating breast cancer-specific textural features from anatomical and functional MR images enhances radiomics performance, offering a noninvasive and effective tool for clinical practice [29] Han et al. further developed a radiomic nomogram for predicting axillary lymph node metastasis in breast cancer patients. This nomogram, combining a radiomic signature with clinical features, showed excellent predictive ability, with AUCs of 0.84 and 0.87 in training and validation sets, respectively. Additionally, another radiomic signature distinguished between cases with fewer than two versus more than two metastatic nodes, with moderate performance (AUC 0.79) [30]. However, the majority of radiomics studies have extracted contrast-enhanced MRI data, and their processing still requires international standardization.

Recently published trials such as INSEMA or SOUND takes further the axillary assessment and change the surgical management in patients with breast cancer [31,32]. Both trials suggest that for certain BC patients (T1, hormone receptor positive, HER2 negative, >50y, etc.) without axillary metastasis, axillary surgery may be safely omitted without compromising survival outcomes.

Thus, it is becoming imperative to find even better, and accurate imaging modalities to non-invasively assess the axilla of BC patients. This study contributes to the literature as the first to evaluate the application of MRI relaxometry in axillary lymph node assessment in BC patients. By quantifying relaxation parameters, this technique offers unique insights into the microstructural differences between metastatic and benign nodes. Notably, the study demonstrated that two MR relaxometry parameters, T2Min-ipsilateral and 1HAv-ipsilateral, are significant predictors of metastatic involvement, with respective AUC values of 0.681 and 0.740. Furthermore, integrating relaxometry parameters with clinical and pathological data, such as Nottingham grade and ER status, resulted in an improved AUC of 0.749. These findings highlight the potential of relaxometry to enhance diagnostic accuracy and provide a more personalized approach to staging and treatment planning.

There are some limitations to note. The retrospective design may introduce selection bias, and the relatively small sample size of 67 patients limits the generalizability of the findings; we did not include the total number of affected lymph nodes. Even if a large number of confounding factors were included in the analysis (such as tumor size, hormonal receptor status), there might be other potential confounding factors, such as genetic status, that were not investigated. These factors could independently affect both MRI relaxometry parameters and axillary lymph node status, potentially distorting the results. Prospective study designs with randomization or matching techniques could help minimize confounding by evenly distributing these factors across groups. Additionally, the absence of direct comparison with alternative advanced imaging techniques, such as PET-CT or radiomics-based MRI, constrains the broader applicability of the results. Future research should focus on prospective, multi-center studies with larger cohorts to validate these results and compare relaxometry with other imaging modalities.

Future research should focus on directly comparing MRI relaxometry with other advanced imaging modalities, such as FDG PET, to evaluate their respective capabilities in predicting axillary metastases. FDG PET, known for its utility in metabolic staging, could serve as a benchmark to highlight the added value or complementary role of MRI relaxometry in clinical practice. Additionally, radiomics provides an exciting opportunity to enhance the diagnostic power of MRI relaxometry. By extracting detailed quantitative imaging features from relaxometry maps, researchers could uncover novel predictors of axillary tumor burden, potentially improving accuracy and risk stratification. These radiomics-driven insights could pave the way for more personalized treatment approaches. Finally, larger, multicenter prospective studies are needed to validate the findings of this study and ensure their generalizability across diverse patient populations.

## 5. Conclusions

The current study demonstrates that MRI relaxometry is a promising non-invasive diagnostic tool for evaluating axillary lymph nodes in breast cancer patients. There was a statistical correlation between the presence of estrogen receptors, more advanced Nottingham grade and axillary lymph node status, and no statistical correlation between patient age, tumor type, presence of progesterone receptors or HER 2 and axillary lymph node status. The MR relaxometry technique achieved good discriminatory performance, particularly when combined with clinical and pathological data, underscoring its potential to complement existing diagnostic workflows. By providing accurate axillary staging, MRI relaxometry has the potential to reduce reliance on axillary ultrasound and invasive biopsies, streamlining the diagnostic process and minimizing patient burden. These findings pave the way for further research into the broader application of relaxometry in breast cancer staging and its integration into routine clinical practice.

## Figures and Tables

**Table 1:** Clinical, histology and immunohistochemistry characteristics of the study group.

	Patients with Axillary Metastasis	Patients without Axillary Metastasis	*p*-Value
Age	55.4 ± 11.58	57.1 ± 10.17	0.481
Histology IDC * ILC	26 3	37 0	
Nottingham grade 1 2 3	4 13 12	16 12 10	0.042
ER + −	18 11	33 5	0.019
PR + −	11 9	28 16	0.165
Ki67% ≥20% <20%	9 20	19 19	0.121
HER2 + −	9 20	5 33	0.076
Total	29	38	67

* IDC = invasive ductal carcinoma; ILC = invasive lobular carcinoma; ER = estrogen receptor; PR = progesterone receptor; HER2 = human epidermal growth factor 2.

**Table 2:** MR relaxometry parameters of ipsilateral and contralateral lymph nodes.

	Patients with Axillary Metastasis	Patients without Axillary Metastasis	*p*-Value
	(*n* = 38)	(*n* = 29)	
Nr.pixel-ipsilateral (pixeli)	1684.44 ± 1352.99 ^a^	2130.00 ± 2441.03	0.381
T2MAX-ipsilateral (ms)	2995.05 ± 1390.52	3229.27 ± 1243.53	0.470
T2MIN-ipsilateral (ms)	41.99 ± 53.30	73.94 ± 53.32	**0.018**
T2AV-ipsilateral (ms)	612.87 ± 271.33	605.82 ± 376.22	0.932
1hMAX-ipsilateral (AH)	484.98 ± 224.46	438.41 ± 250.68	0.434
1hMIN-ipsilateral (AH)	57.08 ± 44.04	59.66 ± 29.35	0.775
1Hav-ipsilateral (AH)	186.79 ± 67.98	148.28 ± 31.60	0.003
Nr.pixel-contralateral (pixeli)	2895.94 ± 1868.23	2577.44 ± 2073.80	0.518
T2MAX-contralateral (ms)	3690.83 ± 722.18	3699.13 ± 745.51	0.963
T2MIN-contralateral (ms)	31.55 ± 51.02	29.13 ± 36.14	0.850
T2AV-contralateral (ms)	631.81 ± 209.51	630.43 ± 244.04	0.980
1hMAX-contralateral (AH)	538.71 ± 262.33	463.45±218.30	0.205
1hMIN-contralateral (AH)	34.07 ± 23.86	42.28 ± 18.42	0.116
1Hav-contralateral (AH)	172.65 ± 26.95	153.37 ± 29.12	**0.007**
^a^ standard deviation; ms = milliseconds; AH = signal units.			

## Data Availability

The original contributions presented in this study are included in the article. Further inquiries can be directed to the corresponding authors.
